# A comparative study on the lipidome of normal knee synovial fluid from humans and horses

**DOI:** 10.1371/journal.pone.0250146

**Published:** 2021-04-16

**Authors:** Marta K. Kosinska, Gerrit Eichner, Gerd Schmitz, Gerhard Liebisch, Jürgen Steinmeyer

**Affiliations:** 1 Department of Orthopaedics, Laboratory for Experimental Orthopaedics, Justus Liebig University Giessen, Giessen, Germany; 2 Mathematical Institute, Justus Liebig University Giessen, Giessen, Germany; 3 Institute of Clinical Chemistry and Laboratory Medicine, University Hospital Regensburg, Regensburg, Germany; Université Clermont Auvergne - Faculté de Biologie, FRANCE

## Abstract

The current limitations in evaluating synovial fluid (SF) components in health and disease and between species are due in part to the lack of data on normal SF, because of low availability of SF from healthy articular joints. Our study aimed to quantify species-dependent differences in phospholipid (PL) profiles of normal knee SF obtained from equine and human donors. Knee SF was obtained during autopsy by arthrocentesis from 15 and 13 joint-healthy human and equine donors, respectively. PL species extracted from SF were quantitated by mass spectrometry whereas ELISA determined apolipoprotein (Apo) B-100. Wilcoxon’s rank sum test with adjustment of scores for tied values was applied followed by Holm´s method to account for multiple testing. Six lipid classes with 89 PL species were quantified, namely phosphatidylcholine, lysophosphatidylcholine, sphingomyelin, phosphatidylethanolamine, plasmalogen, and ceramide. Importantly, equine SF contains about half of the PL content determined in human SF with some characteristic changes in PL composition. Nutritional habits, decreased apolipoprotein levels and altered enzymatic activities may have caused the observed different PL profiles. Our study provides comprehensive quantitative data on PL species levels in normal human and equine knee SF so that research in joint diseases and articular lubrication can be facilitated.

## Introduction

The motion of joints requires the lubrication of articulating surfaces with synovial fluid (SF) that acts as the lubricant occupying the joint cavity and limiting adhesion formation [1, 2]. Amongst its other functions, SF supplies the avascular cartilage with nutrients and oxygen, provides a medium for waste transport, and mediates cell-cell communication through cytokines and growth factors [3]. The measurement of SF in healthy human knee joints is problematic, only being found in small quantities of approx. 2.5 ml from which about 0.5 ml can be aspirated. The SF is spread over the internal joint surfaces to produce a film with varying thicknesses averaging at 24 μm thick. Large animals like horses may have larger volumes of up to 25 ml as are found in equine ankle joints, possibly resulting in part from the effect of gravity on capillary filtration pressure.

The synovial microvasculature is essential for the generation of SF since synovial capillaries are fenestrated with pores covered by a thin membrane [4, 5]. SF is viewed as a passive ultrafiltrate of plasma generated across the walls of the synovial lining capillaries, in which fibroblast-like type B synoviocytes actively secrete additional compounds such as hyaluronan, phospholipids (PLs), and lubricin [3, 6]. The formation of this ultrafiltrate depends on the difference between the intracapillary and intraarticular hydrostatic pressure as well as between the colloid osmotic pressures found within the blood vessels and the SF. The fenestrations observed in the synovial endothelium and the macromolecular hyaluronic acid sieve within the interstitial matrix limits the diffusion of high-molecular-weight solutes into SF.

Plasma proteins, including apolipoprotein (Apo) A1 and Apo B, as well as triglycerides and total cholesterol are also found at reduced concentrations in SF, the levels of which are inversely proportional to their molecular size. For instance, in patients with knee osteoarthritis (OA) the SF/serum concentration ratios for total protein, Apo A1, Apo B, triglycerides and total cholesterol lie at 0.57, 0.29, 0.24, 0.17 and 0.35, respectively [7]. As such, the majority of PLs (with phosphatidylcholine (PC) being the most abundant PL class) appear to derive from the synovial lining capillaries. In addition, local sources can contribute lipids to the SF as they are released from damaged cells or are produced and secreted by, e.g., synovial fibroblasts and chondrocytes [8–10]. Ultrastructural analysis of healthy SF from rat, dog and horse suggest a complex structure of SF consisting of a network of multilamellar vesicles based on a glycoprotein gel and coated with a lipid-based membrane [11]. Finally, SF is cleared by the plexus of lymphatics located within the sub-synovium in a manner which is assisted by joint movement [6]. Smaller molecules such as cytokines, lactate and carbon dioxide can also be cleared by direct diffusion into the synovial capillaries [12].

Over the past two decades, important new knowledge has become known about the various functions of PLs. This has often been related to the boundary lubrication of articular joints as well as to immunological and inflammatory reactions in studies assessing pro-resolving lipid mediators and lipid antigens [1, 2, 13–17]. Moreover, the SF profiles of PL species are modified in human and canine OA knees [18, 19], although disease related changes of PL levels are still poorly understood. Various species often used as models for musculoskeletal diseases like OA such as small rodents or even larger animals like sheep, goat, dog or horse may also be used to study PL functions in OA. However, the anatomy of joints can be quite varied across different species, e.g. with regard to cartilage thickness, the size of cartilage layers, chondrocyte density, and the diffusional and nutritional environment [20, 21]. Interestingly, based on these cartilage parameters, the horse approximates quite closely to man. As two examples, the knee joint cartilage thickness varies from 1.5–2.0 mm in horse and 2.2–2.5 mm in human while the cellularity ranges from 20–40 cells/mm^3^ in horses and 10–30 cells/mm^3^ in man [20–22].

Although larger animals such as the horse appear to simulate the human situation more closely as regards anatomy, articular cartilage and joint loading patterns, data on SF from healthy normal joints is rare from these species. In addition, lubrication in aqueous media such as SF has attracted the interest of many scientists, even though data regarding the complex lipid composition in normal SF is sparse. The current limitations of comparing SF compounds between diseased groups and species can be traced to the lack of data on normal SF and the difficulties experienced when collecting it. Importantly, substantial progress in lipidomic technology has now enabled the quantification of a large variety of lipids even in small volumes of SF. Scientific progress in the understanding and treatment of OA might result from studies on human and equine patients that may also benefit from clinical trials performed in the other species. The aim of the present study was therefore to evaluate species-dependent differences in PL composition of normal SF obtained from the knee joints of equine and human donors. We hypothesize that our quantitative analysis of a large variety of PLs will reveal an equine SF lipid profile that closely resembles that of the human lipid profile.

## Materials and methods

### Chemicals

Sigma (Taufkirchen, Germany) was the supplier of all reagents unless otherwise specified. Chloroform and methanol, both HPLC-grade, as well as neomycin sulfate and gentamycin sulfate were obtained from Merck (Darmstadt, Germany). Avanti Polar Lipids (Alabaster, AL, USA) was the source for the lipid standards.

### Equine synovial fluid

SF was acquired from the knees of 13 horses after medically indicated euthanasia [(7 males, 6 females, median age 14 (11–16) years, median weight 550 (480–578) kg]. Equine SF was only used from healthy knees or those with a low Collins grade of disease [0.375 (0–0.5), n = 13], and with no further history of any arthritic disease. Equine SF was collected by the Equine Clinic (Department of Veterinary Medicine, Justus Liebig University Giessen) and stored at 4°C until it was extracted as described below within 12 h after aspiration. As the used knee SF were obtained from horses which were euthanized on humane grounds for reasons unrelated to the study no ethical approval was necessary according to the German Animal Welfare Act (Tierschutzgesetz) and no special permission of the Local Ethical Committee of the Justus Liebig University Gießen, Germany, was needed.

### Human synovial fluid

SF was acquired during autopsy from the knees of 15 post-mortem human donors without any reported history of arthritic disease [(14 males, 1 female, median age 24.0 (21.0–29.0) years, median BMI 24.8 (20.8–25.0)], as already described [19, 23]. The 15 donors were examined at the Institute of Forensic Medicine, Justus Liebig University Giessen. The Ethical Review Committee of the Faculty of Medicine (Justus Liebig University Giessen, Germany) approved the present study. The Ethical Review Committee (protocol #62/06) waived the need for consent to be obtained from relatives of deceased donors, since a judicial order to perform autopsy existed and any additional emotional draining of the relatives was to be avoided.

### Sampling of synovial fluid

Knee SF was obtained by arthrocentesis as described elsewhere [19]. In brief, SF samples that were contaminated with blood or excessively turbid were excluded by visual inspection. Human SF samples were diluted by addition of 2.0 ml 0.9% NaCl. Human and equine SF were incubated at 37°C for 15 min before filtering with a 1.2-μm filter. After addition of a 10% (v/v) cocktail containing proteases and phospholipase inhibitors, cellular particles were eliminated by centrifugation (16,100 x *g*, 45 min, RT), and subsequently frozen at -86°C until further analysis [19]. For ELISA assays, equine SF samples were incubated with 20 μl hyaluronidase (1U/μl; STEMCELL Technologies, Cologne, Germany) for 15 min. to reduce their viscosity before assaying.

### Analysis of apolipoprotein B

The concentrations of human Apo B 100 and horse Apo B were assessed in SF by ELISA (ApoB100 ELISA Kit from RayBiotech, Norcross, GA, USA for human and the ELISA kit from Cusabio, Houston, TX, USA for horse Apo B) according to the instructions of the manufacturers.

### Phospholipid extraction and mass spectrometric quantification

PLs were extracted together with non-natural lipid species designated to serve as internal standards as described previously [19, 24]. PL and sphingolipid (SL) species were analysed quantitatively on a Quattro Ultima™ triple quadrupole mass spectrometer (Micromass, Manchester, United Kingdom). The analytical procedure including the algorithm used for analysing data obtained by electrospray ionization tandem mass spectrometry (ESI-MS/MS) is described elsewhere [24]. For each lipid class, a self-programmed Microsoft Excel macro was applied for data analysis and to correct for the isotopic overlap of lipid species [24]. This procedure was developed for humans, and was applied here for horse analysis without any further modifications.

The annotation of PL species was performed as described previously for the shorthand notation of lipid structures obtained by mass spectrometry [25]. Glycerophospholipid species were annotated based on the presupposition that the fatty acids only have even numbers of carbon atoms. Sphingomyelin species were assigned based on the assumption of a sphingoid base with 2 hydroxyl groups.

### Data presentation and statistics

**Fig 1:**
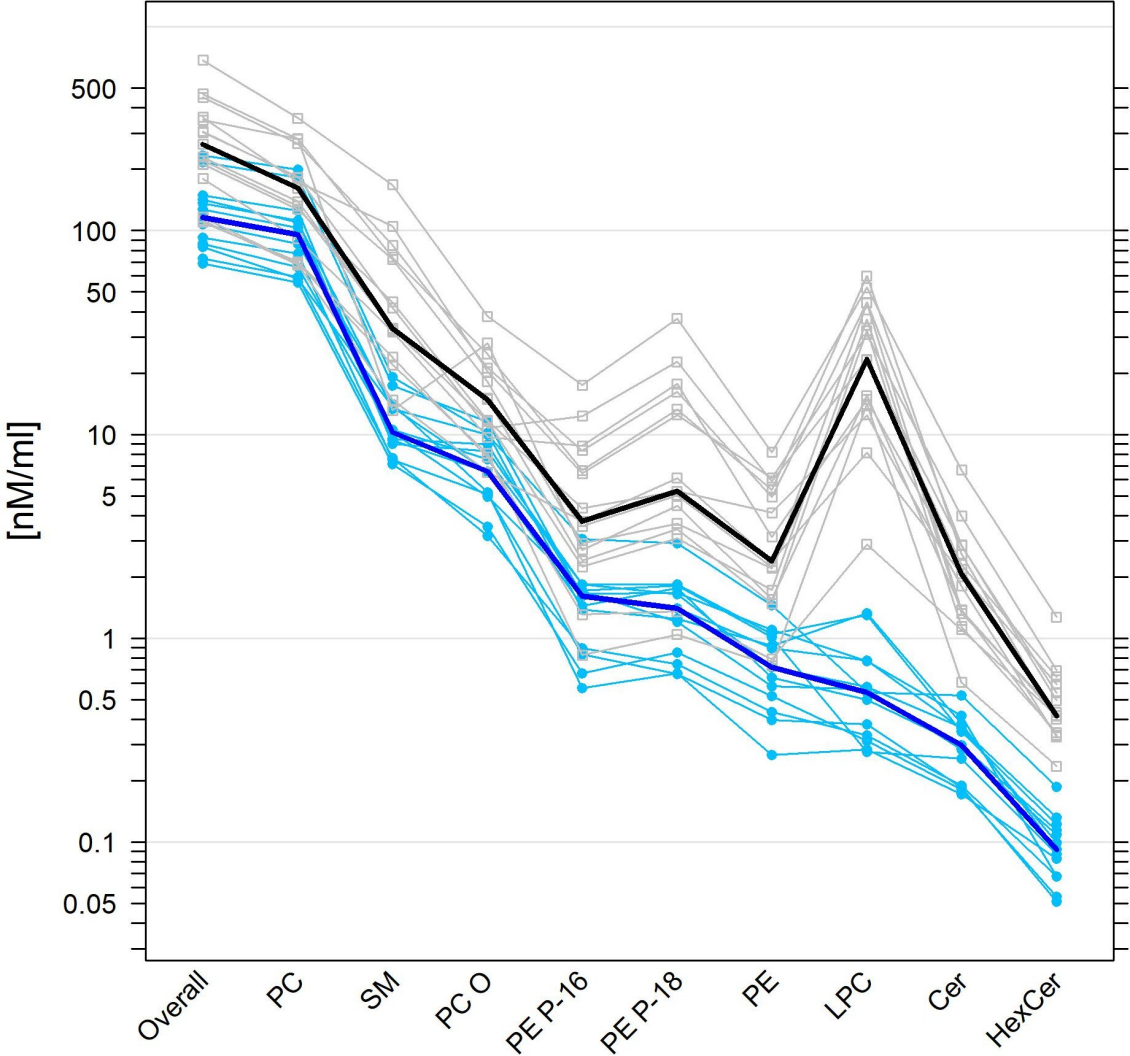
Superposed parallel plots for normal human and equine SF levels of phospholipid classes. SF from human (n = 15) and equine (n = 13) healthy knees were analysed by mass spectrometry. Univariate scatterplots of individual data (human: Grey open squares; equine: Light blue filled circles) of all variables are juxtaposed parallel to each other, here on a log_10_-transformed scale. Values from the same individual are linked by a polyline (colour-coded according to human or equine group membership) thus forming a ‘profile’ for each individual. Median values of the variables across individuals in each group are presented as superposed, thicker profiles (human: Black; equine: Dark blue). PC (O)-phosphatidylcholine + ether phosphatidylcholine, LPC-lysophosphatidylcholine, SM-sphingomyelin, PE-phosphatidylethanolamine, PE P-phosphatidylethanolamine-based plasmalogen, Cer-ceramide, HexCer-hexosyl ceramide.

**Fig 2:**
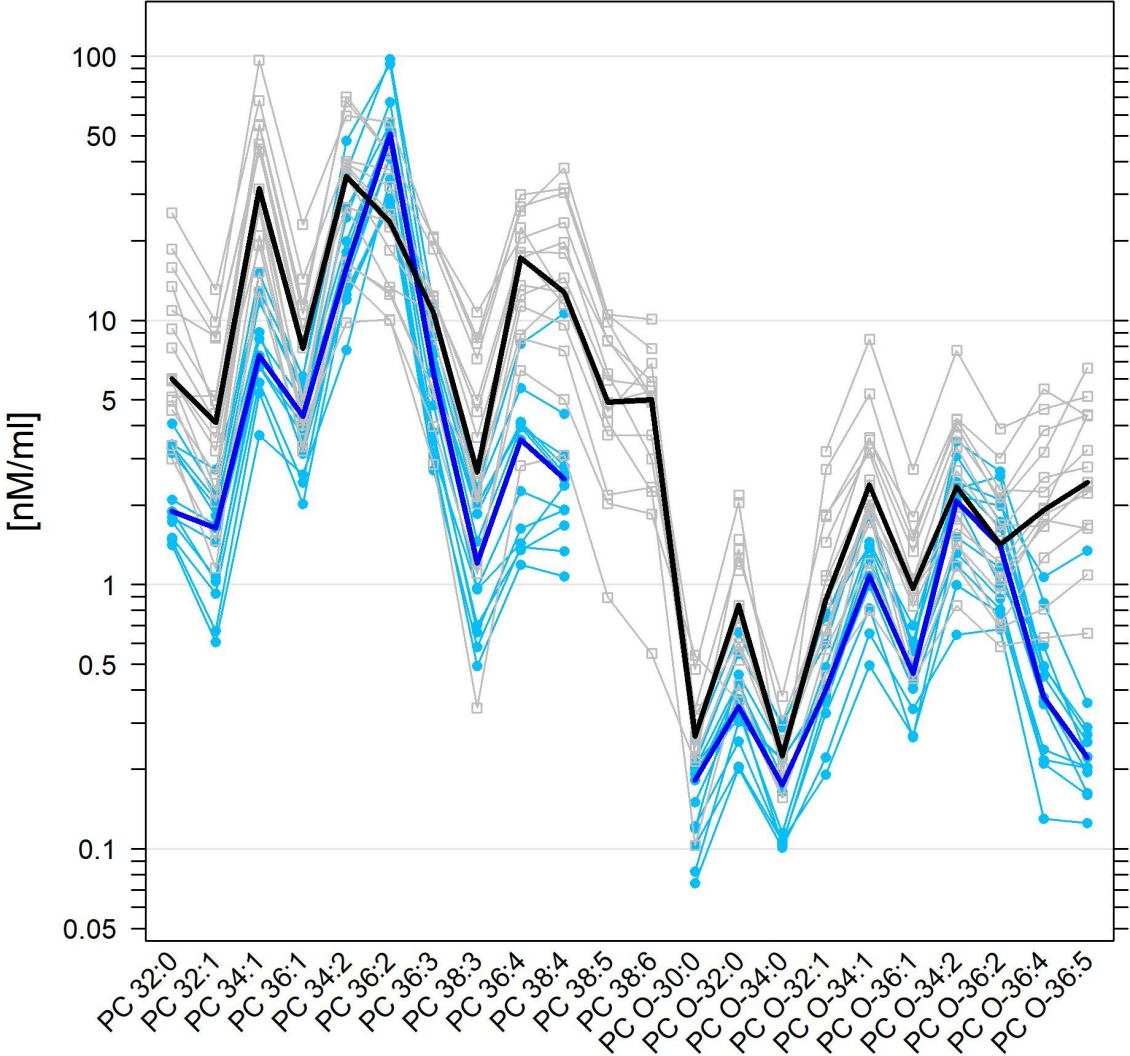
Superposed parallel plots for normal human and equine SF levels of PC (O) species. The univariate scatterplots of PC (O) species concentrations in SF (human: Grey open squares; equine: Light blue filled circles) are juxtaposed parallel to each other, here on a log_10_-transformed scale. Lipid concentration values from the same individual are linked by a polyline (colour-coded according to human or equine group membership) thus forming a ‘profile’ for each individual. Some lipid concentration values are missing due to their levels being below 1% of the total PC (O) class, which interrupt the profile. Median values of lipid species concentrations in SF across individuals in each group are presented as superposed, thicker profiles (human: Black; equine: Dark blue) resulting in group-specific ‘median profiles’.

**Fig 3:**
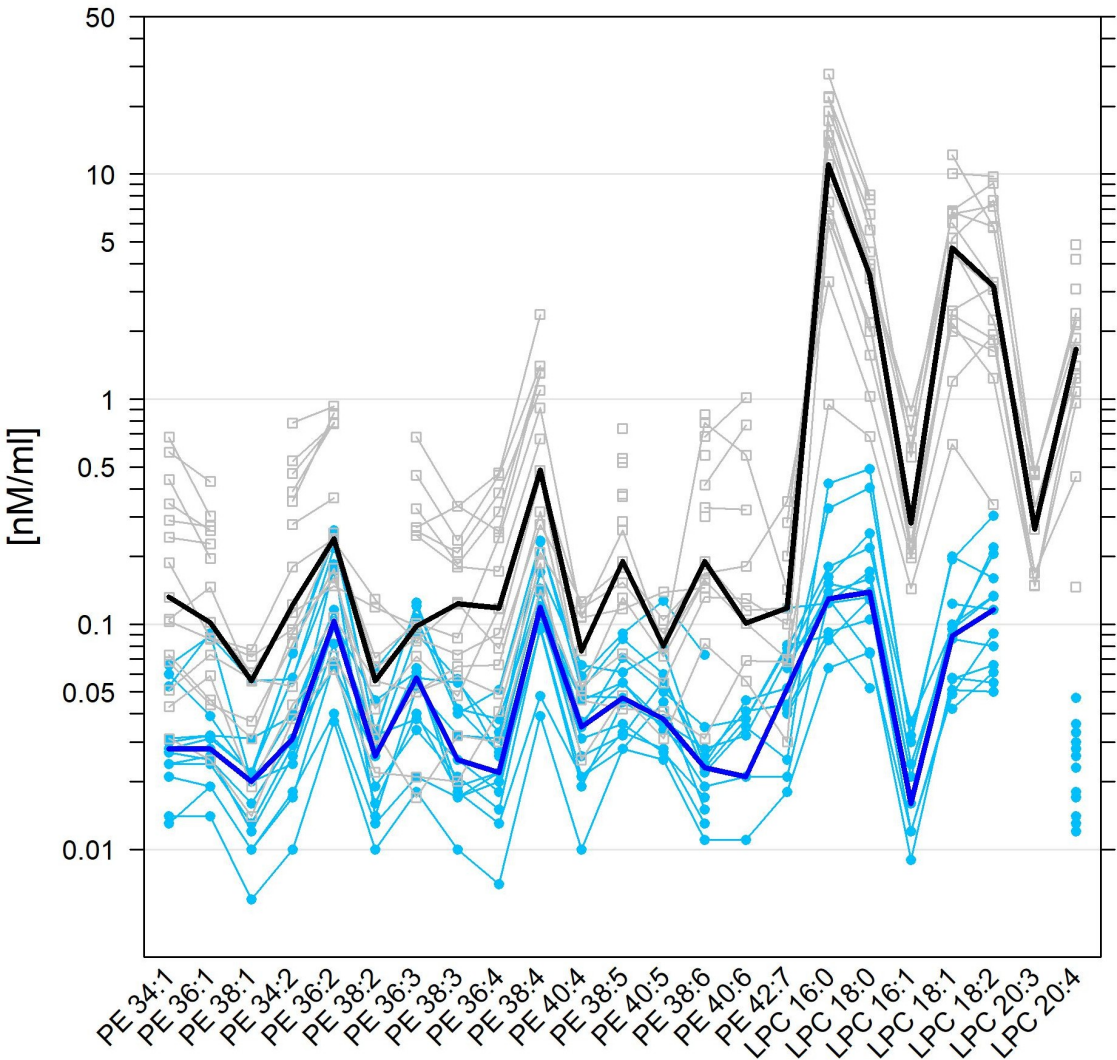
Superposed parallel plots for normal human and equine SF levels of PE and LPC species. For further details, see the caption of Fig 2, which is fully analogous, but here for PE and LPC species.

**Fig 4:**
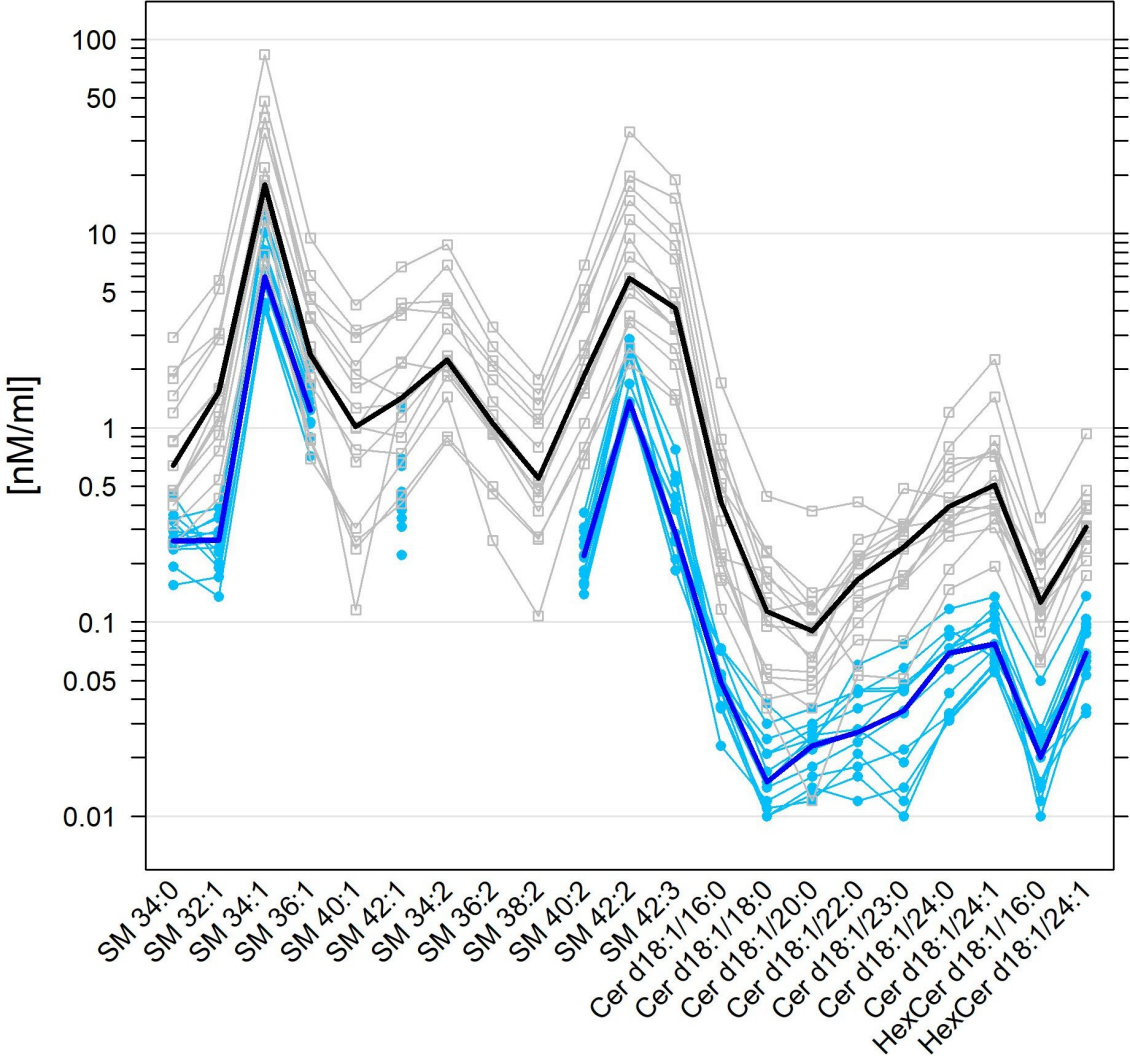
Superposed parallel plots for normal human and equine SF levels of SM and Cer species. For further details, see the caption of Fig 2, which is fully analogous, but here for SM and Cer species.

**Fig 5:**
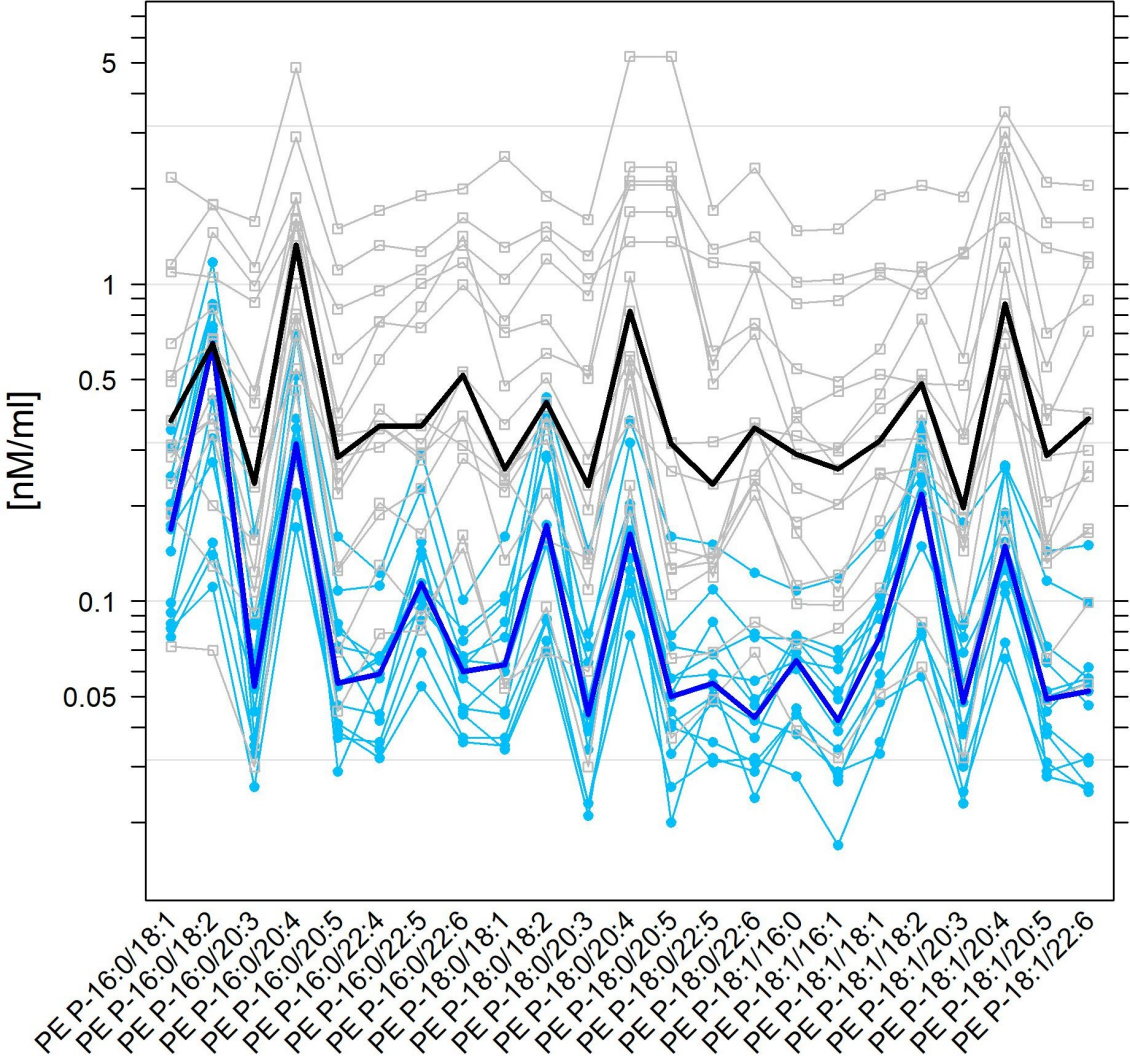
Superposed parallel plots for normal human and equine SF levels of PE P-16 and PE P-18 species. For further details, see the caption of Fig 2, which is fully analogous, but here for PE-P-16 and PE P18 species.

Human and equine lipid concentrations representing at least 1% of their total corresponding lipid class were selected. Within the text, the values are the medians presented with their quartile ranges in parenthesis. For statistical analyses and graphics, the open source software R 3.6.3 [26] together with its lattice add-on package [27] were used. For graphical exploratory presentations, parallel (coordinate) plots [28] of data are presented (Figs 1–5). As descriptive statistics the medians, 1st and 3rd quartiles, and the ratios of human to equine medians are presented (S1 and S2 Tables in S1 File). Wilcoxon’s rank sum test with adjustment of scores for tied values from the R-package coin [29] was applied for lipid classes and species to evaluate whether the distributions of lipid concentrations in humans and horses were displaced relative to each other. To account for multiple testing, we used Holm’s method to adjust the *p*-values in families of lipid classes and species. Statistical significance was declared where *p*-values were at most 0.05.

## Results

### Level of apolipoprotein B-100 in knee SF

We determined Apo B-100 levels in human SF of 11.7 (6.8–16.1) mg/dL and in equine SF of 8.0 (5.8–11.7) mg/dL. Human SF contained a 1.5-fold higher amount of Apo B-100 than equine SF.

### The level and composition of PLs in healthy equine and human SF

In total 89 individual lipid species were detected in SF, and 83 different lipid species belonging to six lipid classes were quantified in both normal equine and human SF (Fig 1, S1 Table in S1 File) with levels above 1% of their corresponding PL class. Of these, 20 or 22 phosphatidylcholine (PC) O-ether PC (PC O) species, 6 or 7 lysophosphatidylcholine (LPC) species, 9 or 12 sphingomyelin species (SM), 16 phosphatidylethanolamine (PE) species, 23 phosphatidylethanolamine-based plasmalogen (PE P) species, and 9 ceramide (Cer) hexosyl ceramide (HexCer) species were quantified in equine or human SF, respectively, with levels above 1% of the corresponding PL class. The concentrations of lipid species can be found in S2 Table in S1 File, which displays the medians, 1^st^ and 3^rd^ quartiles, the ratios of human to equine medians and the results of the statistical analyses.

In order to elucidate the extent to which mammalian species-dependent differences exist between both SF lipidomes, we compared the PL profile in 13 equine SF samples with those of 15 human donors. Equine and human SF were obtained from knee joints without any documented history of joint disorder. In general, we found that the levels of total lipid species in equine SF [116.1 (86.3–141.8) nmol/ml] were approximately half (0.44-fold, P<0.001) those of human SF [265.3 (195.7–354.1) nmol/ml], and that the lipid composition was comparable with PC (O), SM, PE, LPC, PE P and Cer representing the main PL classes.

Even though the level of total PL and the concentrations of the dominant PL class PC were already significantly higher in human SF, the levels of the other PL classes such as SM, PC (O), PE P, LPC, and Cer were further markedly increased in human SF when compared to equine SF (Fig 1). Specifically, we found that the respective median levels of the PC, PC O, LPC, PE, PE P-16, and PE P-18 classes were 1.68-fold (P = 0.019), 2.26-fold (P<0.001), 43.1-fold (P<0.001), 3.31-fold (P<0.001), 2.32-fold (P = 0.001), and 3.75-fold (P<0.001) higher in human than in equine SF (Fig 1, S1 Table in S1 File). Similarly, the respective levels of the SM, Cer, and HexCer classes were markedly elevated in human SF by 3.23-fold (P<0.001), 6.94-fold (P<0.001), and 4.53-fold (P<0.001) compared with equine SF (Fig 1, S1 Table in S1 File).

In addition, most of the PLs were found at lower levels in equine SF compared with human SF, thus reflecting the data obtained for the corresponding class. Interestingly, levels of those lipid species with more than two double bonds were often lower in equine than in human SF. Particularly pertinent were the markedly decreased levels in equine SF for some PC (O) and SM lipid species as well as all LPC species. As one example, PC (O) species with highly polyunsaturated long-chained fatty acids such as PC 36:4, PC O-36:4, PC O-36:5, PC 38:4, PC 38:5, PC 38:6, and PC 40:6 were present at markedly lower levels in equine SF compared to human SF and compared to other PC species (Fig 2, S2 Table in S1 File).

Similarly, our data on PE species with highly polyunsaturated fatty acids such as PE 38:3, PE 36:4, PE 38:4, PE 38:6 and PE 40:6 but not PE 42:7 revealed much higher levels in human compared to equine SF. Fig 3 also demonstrates that all LPC species were markedly elevated in human SF compared to equine SF (S2 Table in S1 File).

Similarly, the concentrations of SM species with more than one double bond, particularly SM 34:2, SM 36:2, SM 38:2, SM 40:2, and SM 42:3, were significantly lower or even below 1% of the total SM class in equine SF when compared to human SF (Fig 4, S2 Table in S1 File). Cer or HexCer species were on average 6.94-fold or 4.53-fold higher in human SF when compared with normal equine SF and all individual species followed this pattern (Fig 4, S1 Table in S1 File).

Again, we detected markedly elevated levels for nearly every PE P species in human SF compared to equine SF. Remarkably, the PE P species with highly polyunsaturated fatty acids such as PE P-16:0/20:5, PE P-16:0/22:4, PE P-16:0/22:6, PE P 18:0/20:5, PE P-18:0/22:6, and PE P-18:1/22:6 were strongly elevated in human SF (Fig 5, S2 Table in S1 File). Even though 29 out of 30 PE P species were detected at markedly decreased levels in equine SF compared to human SF, only the PE P-16:0/18:2 was found at an almost equivalent concentration in equine SF (1.0-fold, P = 0.751).

## Discussion

Articular knee joints of horses are similar to those of humans with regard to their anatomy, thickness and zonal structure of articular cartilage, and loading patterns. However, data on the composition of lipids of SF from healthy normal joints is rare mainly because of the limited availability. Major progress in lipidomic technology has now allowed us to quantify a large variety of PL species within a small SF volume. We found six major PL classes in equine knee SF, namely PC/PC O, LPC, SM, PE, PE P, and Cer, albeit in differing proportions. Here, we report for the first time that compared to healthy human knee SF, equine SF contains only half of the total PL content, and that all PL classes together with most of their species were present at markedly reduced levels.

The diminished total PL content in equine SF compared to human SF is accompanied by a lower level of apolipoproteins (Apo) in equine SF, as indicated by our finding that levels of Apo B-100 are only 40% relative to human SF. Apo function as structural components in lipid transport. Apo A1 was reported to be the major protein in plasma HDL particles and Apo B-100 is the structural protein found in LDL, IDL and VLDL particles both in humans and horses [30–35]. Apo are mainly produced in the liver and their synthesis is controlled primarily by dietary factors and hormones such as insulin and thyroxin [30–32]. The levels of Apo as well as of triacylglycerols and total cholesterol in SF from OA patients are markedly reduced to one third of those found in serum, indicating a low permeability for larger molecules of the fenestrated synovial endothelium and the interstitial tissue between the blood vessel and synovium [7, 36]. As such, the low levels of Apo in equine SF appear to be at least partly responsible for the low levels of PLs relative to human SF.

Using ^31^P-NMR and MALDI-TOF-MS, Fuchs et al. [37] already reported the relative content of nine selected PLs in the SF of patients with rheumatoid arthritis (RA) and of nine healthy or OA horses. Interestingly, they found only traces or no polyunsaturated PC species like PC 36:4, PC 38:4 and PC 38:5 in horse SF whereas traces or moderate levels of these PC species were reported in SF from RA patients. Our quantitative study supports these data since all nine polyunsaturated PC species with 4, 5 or 6 double bonds were determined at low levels compared with either normal human SF or PC species having fewer double bonds.

It has previously been reported that the lubricating properties of PC species depend on their fatty acid chain lengths and the number of double bonds [38, 39]. As such, lipids with longer fatty acid chains and higher levels of unsaturation might have better lubricating abilities. Horses, in contrast to humans, stand most of the day so that their joints are often exposed to constant loads. We speculate that SF containing fewer PC species with long fatty acid chains and more than three double bonds might be regarded as sufficient for providing lubrication to equine articular joints.

Our study shows that PL composition of SF can markedly differ between these two mammalian species, a profile that has also been shown for plasma [40, 41]. Differing PL profiles among vertebrates were also described for erythrocyte membranes and tissues such as the eye, heart and brain, all of which also depend on health status and differences in enzymatic activity [18, 40–46].

One potential reason underlying the species-dependent lipid profiles in our study may have been differences in dietary habits [47]. Humans are in general omnivores while horses are mere herbivores. A slightly increased or decreased serum level of Apo A1 together with an unaltered Apo B level was observed in human dietary groups comparing vegetarians and vegans with omnivores [31, 48]. However, the dietary consumption of animal products correlates positively with the quantity of polyunsaturated long-chained omega-3 (n-3) fatty acids in the lipid profile [49, 50]. Thus, the levels of n-3 polyunsaturated long-chain fatty acids such as eicosapentaenoic acid (C20:5) and docosahexaenoic acid (C22:6) are lower in the plasma of this group than they are in omnivores [49–51]. In addition, the content of cholesterol and saturated fatty acids in vegetarian diets are typically lower than in omnivorous diets. This also contributes to the differences in plasma lipid profiles observed in 3 large human cohort studies comparing omnivores with vegetarians [52]. However, the definitive effect of diets on the PL profile in plasma or SF is not completely known. Studies on dietary supplementation with n-3 long-chain polyunsaturated fatty acids in horses have already shown that this diet is unable to modify experimentally induced synovitis as determined by the synovial fluid level of prostaglandin E2 [53].

The mass spectrometric method used in the present study was established for PL analysis in human samples and was applied here also for equine SF without any further modifications. In addition, several concentrations of PE, PE P, LPC and Cer lay below 0.1 nmol/ml in equine SF and were therefore only detected with a low precision. Due to ethical considerations, normal SF was only obtained postmortem. However, we already reported that our preliminary experiments revealed no relevant changes in the concentration of PL classes and species when plotted against the postmortem sampling time of maximal 3 days [19]. Another limitation of our study was the limited number of donors due to the considerable low availability of SF from normal knee joints.

Fuchs et al. [37] reported no LPC and only a low level of SM in horse SF whereas human SF from RA patients showed low LPC and the highest SM levels. Our data confirm at least in part these semi-quantitative results, since we were only able to quantify six LPC species at low levels and eight SM species at levels that were always below those of normal human SF. The low level of LPC in equine SF appears to be related to a decreased activity of phospholipase A2 (PLA2). This enzyme cleaves the sn-2 fatty acid from the glycerol backbone of PC to generate LPC [54]. Frequently, arachidonic acid is released from polyunsaturated species which is further processed by cyclooxygenases and lipoxygenases to produce eicosanoids such as the pro-inflammatory prostaglandins and leukotrienes [55]. As such, the diminished levels of LPC species are indicative of a reduced PLA2 activity and of a low prostaglandin production thus reflecting a non-inflamed equine synovium.

## Conclusion

Taken together, our study demonstrates quantitative levels of a wide variety of PL species in knee SF of healthy humans and horses. Importantly, our comparison shows that the PL profiles of normal articular knee SF in both species are more or less similar, but that the median levels of PLs in equine SF are markedly lower than those of humans, while there are also some characteristic species-specific differences in PL composition. Our study provides comprehensive quantitative data on PL species found in normal SF. This should facilitate the quest to optimize joint lubrication during tissue regeneration and OA, to identify novel biomarkers, and to assist evaluation of lipid data derived from equine and human patients.

## Supporting information

S1 File (PDF)

## References

[1] Jahn S, Seror J, Klein J. Lubrication of Articular Cartilage. Annu Rev Biomed Eng. 2016; 18: 235–258. 10.1146/annurev-bioeng-081514-123305 27420572

[2] Dėdinaitė A, Wieland DCF, Bełdowski P, Claesson PM. Biolubrication synergy: Hyaluronan—Phospholipid interactions at interfaces. Adv Colloid Interface Sci. 2019; 274: 102050. 10.1016/j.cis.2019.102050 31669714

[3] Hui AY, McCarty WJ, Masuda K, Firestein GS, Sah RL. A systems biology approach to synovial joint lubrication in health, injury, and disease. Wiley Interdiscip Rev Syst Biol Med. 2012; 4: 15–37. 10.1002/wsbm.157 21826801 PMC3593048

[4] Knight AD, Levick JR. Morphometry of the ultrastructure of the blood-joint barrier in the rabbit knee. Q J Exp Physiol. 1984; 69: 271–288. 10.1113/expphysiol.1984.sp002805 6729017

[5] Knight AD, Levick JR, McDonald JN. Relation between trans-synovial flow and plasma osmotic pressure, with an estimation of the albumin reflection coefficient in the rabbit knee. Q J Exp Physiol. 1988; 73: 47–65. 10.1113/expphysiol.1988.sp003122 3347697

[6] Gibson DS, Rooney ME. The human synovial fluid proteome: A key factor in the pathology of joint disease. Proteomics Clin Appl. 2007; 1: 889–899. 10.1002/prca.200700044 21136742

[7] Oliviero F, Lo Nigro A, Bernardi D, Giunco S, Baldo G, Scanu A, et al. A comparative study of serum and synovial fluid lipoprotein levels in patients with various arthritides. Clin Chim Acta. 2012; 413: 303–307. 10.1016/j.cca.2011.10.019 22037510

[8] Sluzalska KD, Liebisch G, Lochnit G, Ishaque B, Hackstein H, Schmitz G, et al. Interleukin-1β affects the phospholipid biosynthesis of fibroblast-like synoviocytes from human osteoarthritic knee joints. Osteoarthr Cartil. 2017; 25: 1890–1899. 10.1016/j.joca.2017.07.011 28736247

[9] Sluzalska KD, Liebisch G, Wilhelm J, Ishaque B, Hackstein H, Schmitz G, et al. Growth factors regulate phospholipid biosynthesis in human fibroblast-like synoviocytes obtained from osteoarthritic knees. Sci Rep. 2017; 7: 13469. 10.1038/s41598-017-14004-9 29044208 PMC5647370

[10] Tsezou A, Iliopoulos D, Malizos KN, Simopoulou T. Impaired expression of genes regulating cholesterol efflux in human osteoarthritic chondrocytes. J Orthop Res. 2010; 28: 1033–1039. 10.1002/jor.21084 20108316

[11] Matei CI, Boulocher C, Boulé C, Schramme M, Viguier E, Roger T, et al. Ultrastructural analysis of healthy synovial fluids in three mammalian species. Microsc Microanal. 2014; 20: 903–911. 10.1017/S1431927614000415 24641871

[12] Simkin PA, Benedict RS. Iodide and albumin kinetics in normal canine wrists and knees. Arthritis Rheum. 1990; 33: 73–79. 10.1002/art.1780330109 2302270

[13] Sorkin R, Kampf N, Dror Y, Shimoni E, Klein J. Origins of extreme boundary lubrication by phosphatidylcholine liposomes. Biomaterials. 2013; 34: 5465–5475. 10.1016/j.biomaterials.2013.03.098 23623226

[14] Kosinska MK, Ludwig TE, Liebisch G, Zhang R, Siebert H-C, Wilhelm J, et al. Articular Joint Lubricants during Osteoarthritis and Rheumatoid Arthritis Display Altered Levels and Molecular Species. PLoS ONE. 2015; 10: e0125192. 10.1371/journal.pone.0125192 25933137 PMC4416892

[15] Serhan CN, Petasis NA. Resolvins and protectins in inflammation resolution. Chem Rev. 2011; 111: 5922–5943. 10.1021/cr100396c 21766791 PMC3192290

[16] Stables MJ, Gilroy DW. Old and new generation lipid mediators in acute inflammation and resolution. Prog Lipid Res. 2011; 50: 35–51. 10.1016/j.plipres.2010.07.005 20655950

[17] Leslie DS, Dascher CC, Cembrola K, Townes MA, Hava DL, Hugendubler LC, et al. Serum lipids regulate dendritic cell CD1 expression and function. Immunology. 2008; 125: 289–301. 10.1111/j.1365-2567.2008.02842.x 18445008 PMC2669133

[18] Kosinska MK, Mastbergen SC, Liebisch G, Wilhelm J, Dettmeyer RB, Ishaque B, et al. Comparative lipidomic analysis of synovial fluid in human and canine osteoarthritis. Osteoarthr Cartil. 2016; 24: 1470–1478. 10.1016/j.joca.2016.03.017 27049029

[19] Kosinska MK, Liebisch G, Lochnit G, Wilhelm J, Klein H, Kaesser U, et al. A lipidomic study of phospholipid classes and species in human synovial fluid. Arthritis Rheum. 2013; 65: 2323–2333. 10.1002/art.38053 23784884

[20] Frisbie DD, Cross MW, McIlwraith CW. A comparative study of articular cartilage thickness in the stifle of animal species used in human pre-clinical studies compared to articular cartilage thickness in the human knee. Vet Comp Orthop Traumatol. 2006; 19: 142–146. 16971996

[21] Hunziker EB. Biologic repair of articular cartilage. Defect models in experimental animals and matrix requirements. Clin Orthop Relat Res. 1999: S135–46. 10546642

[22] van der Kraan PM. Factors that influence outcome in experimental osteoarthritis. Osteoarthr Cartil. 2017; 25: 369–375. 10.1016/j.joca.2016.09.005 27616682

[23] Kosinska MK, Liebisch G, Lochnit G, Wilhelm J, Klein H, Kaesser U, et al. Sphingolipids in human synovial fluid—a lipidomic study. PLoS ONE. 2014; 9: e91769. 10.1371/journal.pone.0091769 24646942 PMC3960152

[24] Liebisch G, Lieser B, Rathenberg J, Drobnik W, Schmitz G. High-throughput quantification of phosphatidylcholine and sphingomyelin by electrospray ionization tandem mass spectrometry coupled with isotope correction algorithm. Biochim Biophys Acta. 2004; 1686: 108–117. 10.1016/j.bbalip.2004.09.003 15522827

[25] Liebisch G, Vizcaíno JA, Köfeler H, Trötzmüller M, Griffiths WJ, Schmitz G, et al. Shorthand notation for lipid structures derived from mass spectrometry. J Lipid Res. 2013; 54: 1523–1530. 10.1194/jlr.M033506 23549332 PMC3646453

[26] R Core Team. A Language and Environemnt for Statistical Computing.; 2019. Available: https://www.R-project.org/. Accessed 28 February 2020.

[27] Sarkar D. Lattice: Trellis Graphics for R. Available: https://CRAN.R-project.org/package=lattice. Accessed 28 February 2020.

[28] Wegman EJ. Hyperdimensional data analysis using parallel coordinates. J Am Stat Assoc. 1990; 85: 664–675.

[29] Hothorn T, Winell H, Hornik K, van de Wiel MA, Zeileis A. Coin: Conditional Inference Procedures in a Permutation Test Framework.; 2019. Available: https://CRAN.R-project.org/package=coin. Accessed 28 February 2020.

[30] Phillips MC. New insights into the determination of HDL structure by apolipoproteins: Thematic review series: high density lipoprotein structure, function, and metabolism. J Lipid Res. 2013; 54: 2034–2048. 10.1194/jlr.R034025 23230082 PMC3708355

[31] Huang Y, Mahley RW. Apolipoprotein E: structure and function in lipid metabolism, neurobiology, and Alzheimer’s diseases. Neurobiol Dis. 2014; 72 Pt A: 3–12. 10.1016/j.nbd.2014.08.025 25173806 PMC4253862

[32] Ogedegbe HO BDW. Lipids, lipoproteins, and apolipoproteins and their disease associations. laboratorymedicine. 2001; 32: 384–389.

[33] Watson TD, Burns L, Love S, Packard CJ, Shepherd J. The isolation, characterisation and quantification of the equine plasma lipoproteins. Equine Vet J. 1991; 23: 353–359. 10.1111/j.2042-3306.1991.tb03737.x 1959526

[34] Watson TD, Burns L, Love S, Packard CJ, Shepherd J. Plasma lipids, lipoproteins and post-heparin lipases in ponies with hyperlipaemia. Equine Vet J. 1992; 24: 341–346. 10.1111/j.2042-3306.1992.tb02852.x 1396507

[35] Souto PC, Fonseca LAd, Orozco AMO, Lopez CJR, Ermita PAN, Carvalho Filho WP de, et al. Acute-Phase Proteins of Healthy Horses and Horses Naturally Affected by Colic Syndrome. J Equine Vet Sci. 2019; 80: 1–4. 10.1016/j.jevs.2019.06.002 31443825

[36] Levick JR. Synovial fluid and trans-synovial flow in stationary and moving joints. In: Helminen HJ, editor. Joint loading. Biology and health of articular structures. Bristol: Wright; 1987. pp. 149–186.

[37] Fuchs B, Bondzio A, Wagner U, Schiller J. Phospholipid compositions of sera and synovial fluids from dog, human and horse: a comparison by 31P-NMR and MALDI-TOF MS. J Anim Physiol Anim Nutr (Berl). 2009; 93: 410–422. 10.1111/j.1439-0396.2008.00822.x 18484967

[38] Chen Y, Crawford RW, Oloyede A. Unsaturated phosphatidylcholines lining on the surface of cartilage and its possible physiological roles. J Orthop Surg Res. 2007; 2: 14. 10.1186/1749-799X-2-14 17718898 PMC2000865

[39] Trunfio-Sfarghiu A-M, Berthier Y, Meurisse M-H, Rieu J-P. Role of nanomechanical properties in the tribological performance of phospholipid biomimetic surfaces. Langmuir. 2008; 24: 8765–8771. 10.1021/la8005234 18620439

[40] Nouri-Sorkhabi MH, Agar NS, Sullivan DR, Gallagher C, Kuchel PW. Phospholipid composition of erythrocyte membranes and plasma of mammalian blood including Australian marsupials; quantitative 31P NMR analysis using detergent. Comp Biochem Physiol B, Biochem Mol Biol. 1996; 113: 221–227. 10.1016/0305-0491(95)02011-x 8653579

[41] Subbaiah PV, Liu M. Comparative studies on the substrate specificity of lecithin:cholesterol acyltransferase towards the molecular species of phosphatidylcholine in the plasma of 14 vertebrates. J Lipid Res. 1996; 37: 113–122. 8820107

[42] Pamplona R, Portero-Otín M, Ruiz C, Gredilla R, Herrero A, Barja G. Double bond content of phospholipids and lipid peroxidation negatively correlate with maximum longevity in the heart of mammals. Mech Ageing Dev. 2000; 112: 169–183. 10.1016/s0047-6374(99)00045-7 10687923

[43] Moesgaard B, Petersen G, Mortensen SA, Hansen HS. Substantial species differences in relation to formation and degradation of N-acyl-ethanolamine phospholipids in heart tissue: an enzyme activity study. Comp Biochem Physiol B, Biochem Mol Biol. 2002; 131: 475–482. 10.1016/s1096-4959(02)00003-9 11959029

[44] Maldjian A, Cristofori C, Noble RC, Speake BK. The fatty acid composition of brain phospholipids from chicken and duck embryos. Comp Biochem Physiol B, Biochem Mol Biol. 1996; 115: 153–158. 10.1016/0305-0491(96)00086-7 8938995

[45] Panz T, Lepiarczyk M, Zuber A. Comparing the content of lipids derived from the eye lenses of various species. Folia Histochem Cytobiol. 2011; 49: 425–430. 10.5603/fhc.2011.0060 22038221

[46] Ferlazzo AM, Bruschetta G, Di Pietro P, Medica P, Notti A, Rotondo E. Phospholipid composition of plasma and erythrocyte membranes in animal species by 31P NMR. Vet Res Commun. 2011; 35: 521–530. 10.1007/s11259-011-9496-4 21881904

[47] Huang T, Yu X, Shou T, Wahlqvist ML, Li D. Associations of plasma phospholipid fatty acids with plasma homocysteine in Chinese vegetarians. Br J Nutr. 2013; 109: 1688–1694. 10.1017/S000711451200356X 22935202

[48] Lee HY, Woo J, Chen ZY, Leung SF, Peng XH. Serum fatty acid, lipid profile and dietary intake of Hong Kong Chinese omnivores and vegetarians. Eur J Clin Nutr. 2000; 54: 768–773. 10.1038/sj.ejcn.1601089 11083485

[49] Kahleova H, Matoulek M, Bratova M, Malinska H, Kazdova L, Hill M, et al. Vegetarian diet-induced increase in linoleic acid in serum phospholipids is associated with improved insulin sensitivity in subjects with type 2 diabetes. Nutr Diabetes. 2013; 3: e75. 10.1038/nutd.2013.12 23775014 PMC3697401

[50] Mann N, Pirotta Y, O’Connell S, Li D, Kelly F, Sinclair A. Fatty acid composition of habitual omnivore and vegetarian diets. Lipids. 2006; 41: 637–646. 10.1007/s11745-006-5014-9 17069347

[51] Rosell MS, Lloyd-Wright Z, Appleby PN, Sanders TAB, Allen NE, Key TJ. Long-chain n-3 polyunsaturated fatty acids in plasma in British meat-eating, vegetarian, and vegan men. Am J Clin Nutr. 2005; 82: 327–334. 10.1093/ajcn.82.2.327 16087975

[52] Yokoyama Y, Levin SM, Barnard ND. Association between plant-based diets and plasma lipids: a systematic review and meta-analysis. Nutr Rev. 2017; 75: 683–698. 10.1093/nutrit/nux030 28938794 PMC5914369

[53] Ross-Jones TN, McIlwraith CW, Kisiday JD, Hess TM, Hansen DK, Black J. Influence of an n-3 long-chain polyunsaturated fatty acid-enriched diet on experimentally induced synovitis in horses. J Anim Physiol Anim Nutr (Berl). 2016; 100: 565–577. 10.1111/jpn.12359 26189710

[54] Ditz T, Schnapka-Hille L, Noack N, Dorow J, Ceglarek U, Niederwieser D, et al. Phospholipase A2 products predict the hematopoietic support capacity of horse serum. Differentiation. 2019; 105: 27–32. 10.1016/j.diff.2018.12.002 30554008

[55] Dennis EA, Norris PC. Eicosanoid storm in infection and inflammation. Nat Rev Immunol. 2015; 15: 511–523. 10.1038/nri3859 26139350 PMC4606863

