# Supplementary material for: A comparative study on the lipidome of normal knee synovial fluid from humans and horses
Source: PLoS One. 2021 Apr 16;16(4):e0250146. doi: 10.1371/journal.pone.0250146 (PMC8051782; doi:10.1371/journal.pone.0250146)
Supplement: S1 File — (PDF) [file pone.0250146.s001.pdf]

S1 File.

A Comparative Study on the Lipidome of Normal Knee Synovial Fluid from Humans and Horses

Marta K. Kosinska[1], Gerrit Eichner[2], Gerd Schmitz[3], Gerhard Liebisch[3], and Juergen Steinmeyer[1*]

1 Laboratory for Experimental Orthopaedics, Department of Orthopaedics, Justus Liebig University Giessen, Giessen, Germany; M.Kosinska@gmail.com; Juergen.Steinmeyer@ortho.med.uni-giessen.de
2 Mathematical Institute, Justus Liebig University Giessen, Giessen, Germany; Gerrit.Eichner@math.uni-giessen.de
3 Department of Clinical Chemistry and Laboratory Medicine, University Hospital Regensburg, Regensburg, Germany; Gerhard.Liebisch@klinik.uni-regensburg.de; Gerd.Schmitz@klinik.uni-regensburg.de

* Correspondence: Juergen.Steinmeyer@ortho.med.uni-giessen.de

Table S1. Human and equine lipid class concentrations in normal knee synovial fluid.

Lipid class	Synovial Fluid [nmol/ml] Horse			Human			Median Human/ Horse	Holm adjusted p-values
	Median	Q1	Q3	Median	Q1	Q3		
**PC**	95.73	65.91	112.0	161.2	109.1	224.7	1.68	0.019
**PC O**	6.56	5.07	8.89	14.81	10.22	20.74	2.26	<0.001
**LPC**	0.54	0.33	0.77	23.35	14.44	40.38	43.1	<0.001
**SM**	10.27	8.96	13.66	33.12	27.89	73.07	3.23	<0.001
**Cer**	0.30	0.19	0.37	2.01	1.35	2.72	6.94	<0.001
**HexCer**	0.09	0.07	0.11	0.42	0.37	0.57	4.53	<0.001
**PE**	0.72	0.52	1.01	2.39	1.64	5.14	3.31	<0.001
**PE P-16**	1.62	0.89	1.71	3.74	2.56	7.49	2.32	0.001
**PE P-18**	1.40	0.85	1.76	5.25	3.54	14.70	3.75	<0.001
**Overall**	116.1	86.3	141.8	265.3	195.7	354.1	2.29	0.001

Lipid species were quantified in knee SF by ESI-MS/MS as described in Materials and Methods. Values presented are medians together with the interquartile ranges and the ratios of median SF concentrations in humans to horses. Data represent the levels (nM/ml) of lipid classes in SF. Q1: 1st Quartil, Q3: 3rd Quartil, PC-phosphatidylcholine, PC O-ether phosphatidylcholine, LPC-lysophosphatidylcholine, SM-sphingomyelin, PE-phosphatidylethanolamine, PE P-phosphatidylethanolamine-based plasmalogen, Cer-ceramide, HexCer-hexosyl ceramide.

Table S2. Human and equine lipid species concentrations in knee synovial fluid.

Lipid specie	Horse SF [nmol/ml]			Human SF [nmol/ml]			Median Human/ Horse	Holm adjusted p-values
	Median	Q1	Q3	Median	Q1	Q3		
**PC 32:0**	1.89	1.73	3.32	6.01	5.06	12.17	3.18	<0.001
**PC 32:1**	1.64	1.03	1.99	4.11	2.44	6.89	2.50	<0.001
**PC 34:1**	7.37	6.68	11.70	31.52	23.69	50.60	4.28	<0.001
**PC 36:1**	4.35	3.13	5.28	7.87	4.17	9.86	1.81	0.026
**PC 34:2**	15.86	13.03	24.53	35.14	21.65	39.60	2.22	0.026
**PC 36:2**	50.87	34.16	55.46	23.46	13.12	34.72	0.46	0.005
**PC 36:3**	6.31	3.51	7.44	10.64	7.15	15.53	1.69	0.016
**PC 38:3**	1.21	0.70	1.99	2.67	2.09	6.08	2.20	0.007
**PC 36:4**	3.55	1.43	4.08	17.25	10.09	21.49	4.86	<0.001
**PC 38:4**	2.52	1.91	2.80	12.79	10.56	21.62	5.08	<0.001
**PC 38:5**	n.a.	n.a.	n.a.	4.90	3.90	8.37	n.a.	n.a.
**PC 38:6**	n.a.	n.a.	n.a.	5.01	2.67	5.75	n.a.	n.a.
**PC O-30:0**	0.18	0.12	0.20	0.27	0.22	0.34	1.47	0.006
**PC O-32:0**	0.35	0.30	0.43	0.83	0.59	1.31	2.4	<0.001
**PC O-34:0**	0.17	0.11	0.22	0.22	0.18	0.26	1.3	0.319
**PC O-32:1**	0.40	0.36	0.49	0.88	0.66	1.63	2.2	<0.001
**PC O-34:1**	1.08	0.99	1.40	2.38	1.90	3.40	2.2	0.001
**PC O-36:1**	0.46	0.40	0.56	0.97	0.67	1.39	2.1	0.002
**PC O-34:2**	2.08	1.31	2.44	2.35	1.55	3.83	1.1	0.510
**PC O-36:2**	1.40	0.89	1.99	1.42	0.99	2.08	1.0	0.860
**PC O-36:4**	0.38	0.24	0.49	1.92	1.66	2.86	5.1	<0.001
**PC O-36:5**	0.22	0.20	0.27	2.44	1.95	4.36	11.0	<0.001
**LPC 16:0**	0.13	0.09	0.16	11.02	6.44	18.11	84.8	<0.001
**LPC 18:0**	0.14	0.11	0.22	3.56	2.09	5.07	25.6	<0.001
**LPC 16:1**	0.02	0.01	0.02	0.28	0.21	0.61	17.62	<0.001
**LPC 18:1**	0.09	0.06	0.10	4.70	2.27	6.71	52.76	<0.001
**LPC 18:2**	0.12	0.07	0.16	3.17	1.88	6.52	27.4	<0.001
**LPC 20:3**	n.a.	n.a.	n.a.	0.26	0.15	0.46	n.a.	<0.001

**LPC 20:4**	0.03	0.02	0.03	1.66	1.16	2.30	63.8	<0.001
**SM 34:0**	0.26	0.24	0.33	0.64	0.45	1.33	2.44	<0.001
**SM 32:1**	0.26	0.20	0.29	1.54	1.00	2.94	5.84	<0.001
**SM 34:1**	5.98	5.37	7.87	17.74	14.55	36.10	2.97	<0.001
**SM 36:1**	1.23	1.05	1.55	2.39	1.74	4.17	1.94	0.009
**SM 40:1**	n.a.	n.a.	n.a.	1.01	0.48	1.99	n.a.	n.a.
**SM 42:1**	0.43	0.38	0.63	1.43	0.82	2.98	3.32	<0.001
**SM 34:2**	n.a.	n.a.	n.a.	2.23	1.90	4.20	n.a.	n.a.
**SM 36:2**	n.a.	n.a.	n.a.	1.05	0.93	1.92	n.a.	n.a.
**SM 38:2**	n.a.	n.a.	n.a.	0.55	0.41	1.08	n.a.	n.a.
**SM 40:2**	0.22	0.18	0.27	1.85	1.28	3.41	8.41	<0.001
**SM 42:2**	1.37	1.25	2.46	5.89	4.33	13.27	8.94	<0.001
**SM 42:3**	0.29	0.26	0.44	4.11	2.88	8.02	14.4	<0.001
**Cer d18:1/16:0**	0.05	0.04	0.05	0.42	0.21	0.58	8.5	<0.001
**Cer d18:1/18:0**	0.02	0.01	0.02	0.11	0.05	0.17	7.5	<0.001
**Cer d18:1/20:0**	0.02	0.02	0.03	0.09	0.05	0.12	3.9	<0.001
**Cer d18:1/22:0**	0.03	0.02	0.04	0.17	0.11	0.21	6.2	<0.001
**Cer d18:1/23:0**	0.04	0.02	0.05	0.24	0.16	0.31	7.0	<0.001
**Cer d18:1/24:0**	0.07	0.03	0.07	0.39	0.31	0.59	5.7	<0.001
**Cer d18:1/24:1**	0.08	0.06	0.10	0.51	0.38	0.77	6.6	<0.001
**HexCer d18:1/16:0**	0.02	0.02	0.03	0.13	0.11	0.18	6.3	<0.001
**HexCer d18:1/24:1**	0.07	0.06	0.09	0.31	0.27	0.39	4.5	<0.001
**PE 34:1**	0.03	0.02	0.03	0.13	0.07	0.32	4.7	<0.001
**PE 36:1**	0.03	0.02	0.03	0.10	0.07	0.24	3.6	0.001
**PE 38:1**	0.02	0.01	0.02	0.06	0.03	0.06	2.8	0.041
**PE 34:2**	0.03	0.02	0.06	0.12	0.09	0.37	3.9	<0.001
**PE 36:2**	0.10	0.07	0.19	0.24	0.15	0.78	2.3	0.053
**PE 38:2**	0.03	0.02	0.03	0.56	0.03	0.07	2.1	0.041
**PE 36:3**	0.06	0.04	0.09	0.10	0.06	0.27	1.7	0.091

**PE 38:3**	0.03	0.02	0.04	0.12	0.06	0.20	4.9	<0.001
**PE 36:4**	0.02	0.02	0.03	0.12	0.07	0.29	5.4	<0.001
**PE 38:4**	0.12	0.10	0.14	0.48	0.30	1.20	4.1	<0.001
**PE 40:4**	0.04	0.02	0.05	0.08	0.05	0.11	2.2	0.041
**PE 38:5**	0.05	0.03	0.06	0.19	0.12	0.37	4.0	<0.001
**PE 40:5**	0.04	0.03	0.06	0.08	0.06	0.09	2.1	0.091
**PE 38:6**	0.02	0.02	0.03	0.19	0.15	0.49	8.3	<0.001
**PE 40:6**	0.02	0.00	0.04	0.10	0.00	0.25	4.8	0.091
**PE 42:7**	0.05	0.04	0.07	0.12	0.07	0.20	2.3	0.041
**PE P-16:0/18:1**	0.17	0.09	0.20	0.37	0.26	0.58	2.20	0.003
**PE P-16:0/18:2**	0.65	0.27	0.85	0.65	0.36	0.95	1.00	0.751
**PE P-16:0/20:3**	0.05	0.04	0.07	0.24	0.14	0.67	4.37	<0.001
**PE P-16:0/20:4**	0.31	0.27	0.38	1.33	0.74	1.74	4.23	<0.001
**PE P-16:0/20:5**	0.06	0.04	0.08	0.28	0.17	0.49	5.16	<0.001
**PE P-16:0/22:4**	0.06	0.04	0.07	0.36	0.26	0.76	6.05	<0.001
**PE P-16:0/22:5**	0.11	0.10	0.14	0.36	0.25	0.93	3.13	0.002
**PE P-16:0/22:6**	0.06	0.05	0.07	0.52	0.35	1.25	8.60	<0.001
**PE P-18:0/18:1**	0.06	0.04	0.09	0.26	0.23	0.73	4.14	<0.001
**PE P-18:0/18:2**	0.17	0.09	0.29	0.42	0.27	0.99	2.45	0.013
**PE P-18:0/20:3**	0.04	0.03	0.06	0.23	0.13	0.73	5.27	<0.001
**PE P-18:0/20:4**	0.16	0.13	0.20	0.82	0.51	1.87	5.05	<0.001
**PE P-18:0/20:5**	0.05	0.04	0.06	0.31	0.13	1.87	6.28	<0.001
**PE P-18:0/22:5**	0.06	0.05	0.07	0.23	0.13	0.59	4.25	<0.001
**PE P-18:0/22:6**	0.04	0.03	0.06	0.35	0.24	0.94	8.19	<0.001
**PE P-18:1/16:0**	0.07	0.04	0.08	0.29	0.14	0.47	4.48	<0.001
**PE P-18:1/16:1**	0.04	0.03	0.06	0.26	0.11	0.48	6.21	<0.001
**PE P-18:1/18:1**	0.08	0.06	0.10	0.32	0.22	0.57	4.17	<0.001
**PE P-18:1/18:2**	0.22	0.08	0.30	0.48	0.27	0.85	2.22	0.012
**PE P-18:1/20:3**	0.05	0.03	0.07	0.20	0.15	0.53	4.10	<0.001

**PE P-18:1/20:4**	0.15	0.11	0.19	0.87	0.53	2.07	5.82	<0.001
**PE P-18:1/20:5**	0.05	0.04	0.06	0.29	0.14	0.62	5.82	<0.001
**PE P-18:1/22:6**	0.05	0.03	0.06	0.38	0.21	1.03	7.23	<0.001

Lipid species were quantified in knee SF by ESI-MS/MS as described in Materials and Methods. Values presented are medians together with the interquartile ranges and the ratios of the median SF concentrations in humans to those of horses. Data represent the levels (nM/ml) of lipid species in SF. IQR: Interquartile range, Q1: 1st Quartil, Q3: 3rd Quartil, PC-phosphatidylcholine, PC O-ether phosphatidylcholine, LPC-lysophosphatidylcholine, SM-sphingomyelin, PE-phosphatidylethanolamine, PE P-phosphatidylethanolamine-based plasmalogen, Cer-ceramide, HexCer-hexosyl ceramide, n.a.- not analysed and shown since values are below 1% of the corresponding PL class.
